# Prognostic value and relapse pattern of HER2‐low in hormone receptor‐positive breast cancer

**DOI:** 10.1111/1759-7714.15221

**Published:** 2024-01-25

**Authors:** Tong Wei, Yikun Kang, Xue Wang, Jian Yue, Binghe Xu, Peng Yuan

**Affiliations:** ^1^ Department of VIP Medical, National Cancer Center/National Clinical Research Center for Cancer/Cancer Hospital Chinese Academy of Medical Sciences and Peking Union Medical College Beijing China; ^2^ Department of Medical Oncology, National Cancer Center/National Clinical Research Center for Cancer/Cancer Hospital Chinese Academy of Medical Sciences and Peking Union Medical College Beijing China; ^3^ Department of Oncology, Beijing Hospital, National Center of Gerontology, Institute of Geriatric Medicine Chinese Academy of Medical Sciences Beijing China

**Keywords:** breast cancer, HER2‐low, HER2‐zero, prognosis, relapse

## Abstract

**Background:**

A new concept of HER2‐low has emerged in recent years. However, the prognostic value and the relapse pattern of HER2‐low is unclear.

**Methods:**

Our study included patients diagnosed with HER2‐negative/hormone receptor‐positive breast cancer to explore the differences in survival outcomes between the HER2‐low group and the HER2‐zero group. More importantly, we explored different recurrence patterns, including the comparison of metastatic sites and recurrence time curve between the two groups.

**Results:**

A total of 797 patients with hormone receptor‐positive breast cancer were analyzed. Similar disease‐free survival (DFS) was observed between the HER2‐low group and HER2‐zero group (HR 0.84, 95% CI: 0.61–1.16, *p* = 0.290). There was also no significant difference in OS between the HER2‐low group and the HER2‐zero group (HR 0.77, 95% CI: 0.46–1.28, *p* = 0.310). When IHC 1+ and 0 were taken as a group, the IHC 2+ group had significantly better DFS than the IHC 1+ and 0 group in some subgroups. The risk of bone metastasis in patients with HER2 IHC 1+ and 0 was significantly higher than that of patients with HER2 IHC 2+ (12.7% vs. 4.7%, *p* < 0.001). Compared with the HER2‐zero group, we found that the HER2‐low group had a more obvious peak in mortality at the time of postoperative 80th–100th month.

**Conclusions:**

No significant difference in DFS and OS between the HER2‐low group and the HER2‐zero group was observed. Patients with HER2 IHC 1+ and 0 tend to develop bone metastasis. The HER2‐low group had a more obvious second peak in mortality.

## Graphical abstract

No significant difference in disease‐free survival (DFS) and overall survival (OS) between the HER2‐low group and the HER2‐zero group was observed. The HER2‐low group had a more obvious second peak in mortality. Patients with HER2 IHC 1+ and 0 tend to develop bone metastasis.

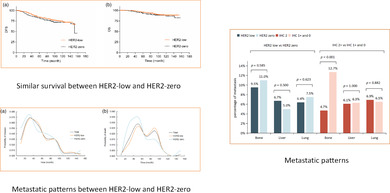

## INTRODUCTION

Among women worldwide, breast cancer is the malignant tumor with the highest incidence.^1^, ^2^ Human epithelial growth factor receptor‐2 (HER2, also known as ERBB2) is an important biomarker that affects breast cancer prognosis and treatment decisions.^3^ Approximately 80% to 85% of patients are HER2‐negative and do not qualify for conventional anti‐HER2 therapy.^4^ A new paradigm of HER2 classification has emerged in recent years based on the rapid development of HER2‐targeted antibody‐drug conjugates (ADCs). For example, trastuzumab‐deruxtecan (T‐DXd) has shown efficacy in patients with HER2‐low breast cancer, defined as HER2 immunohistochemistry (IHC) 1+ or 2+/situ hybridization (ISH) negative, which was recognized as HER2‐negative in the past.^5^

At present, the relationship between HER2‐low and prognosis in breast cancer is controversial. Some studies^6^, ^7^ have found that compared with HER2‐zero, HER2‐low is associated with treatment response and prognosis in breast cancer. However, in other studies,^8^, ^9^, ^10^ the prognosis differences between HER2‐low and HER2‐zero are thought to be not significant and influenced by hormone receptor status.

What is more important is that previous studies mainly focused on the analysis of survival differences with a lack of comparison of recurrence pattern with HER2‐low and HER2‐zero breast cancer, such as the difference of metastatic sites and the difference of the recurrence time curves between HER2‐low and HER2‐zero. Research on the recurrence pattern of HER2‐low and HER2‐zero will help to further understand the heterogeneity of traditionally defined HER2‐negative breast cancer, which will assist in improving the treatment and prognosis of hormone receptor‐positive breast cancer.

In this study, on the one hand, we aimed to explore differences in clinical and pathological features and survival outcomes between the HER2‐low group and the HER2‐zero group in hormone receptor‐positive breast cancer. On the other hand, we explored different recurrence patterns which was rarely studied before, including the comparison of metastatic sites and recurrence time curve between HER2‐low and HER2‐zero in hormone receptor‐positive breast cancer.

## METHODS

### Study design

Our study included 797 patients who were diagnosed with HER2‐negative/hormone receptor‐positive breast cancer from 2010 to 2016 at the Cancer Hospital, Chinese Academy of Medical Sciences. HER2‐low was defined as those with an IHC score of 1+ or 2+ with negative ISH results, and HER2 zero was defined as those with IHC 0. For ease of description, the HER2 IHC 2+ mentioned in the following text stands for IHC 2+ with negative ISH. The inclusion criteria were: (1) Classified as HER2 negative and had available HER2 IHC results. (2) Postoperative pathology showed positive lymph nodes. (3) Perform adjuvant therapy after the surgery. Exclusion criteria were: (1) Stage IV when diagnosed. (2) HER2 IHC 2+ without documented negative ISH results.

### Definitions and outcomes

In this study, the primary outcome was (1) disease‐free survival (DFS), which was defined as the time between randomization and occurrence of local recurrence, regional relapse, distant metastasis, or death from any cause and (2) overall survival (OS), defined as the time between randomization and death from any cause.

### Statistical analysis

Descriptive statistics were used to compare the baseline characteristics of the HER2‐low group and the HER2‐zero group, respectively. The median age was compared using the *t*‐ test. And the other baseline characteristics of the HER2‐low group and the HER2‐zero group were compared using the chi‐square test. Survival was estimated using the Kaplan–Meier curves and compared with a log‐rank test. Cox proportional hazards regression analyses were used to identify the association between survival outcomes, of which the findings were showed as hazard ratios (HRs) with 95% confidence intervals (CIs). All *p*‐values were two‐sided and *p* < 0.05 was considered statistically significant. Statistical analyses were performed using SPSS Statistics version 29.0 (IBM Corporation) and the GraphPad Prism program, version 7.0 (GraphPad Software). The rate of recurrence in a given period was calculated by dividing the number of patients who had disease in a given period by the number of total patients in the group at the beginning. The mortality rate in a given period was calculated by dividing the number of patients who died in a given period by the number of total patients in the group at the beginning.

## RESULTS

### Patient characteristics

A total of 797 women with hormone receptor‐positive breast cancer were enrolled in this study. Among the hormone receptor‐positive group (*n* = 797), there were 597 (74.9%) patients in the HER2‐low group and 200 (25.1%) patients in the HER2‐zero group, respectively. The median follow‐up period was 97.3 (IQR 25%–75%, 68.5–116.4) months.

The baseline clinical and pathological characteristics of HER2‐low and HER2‐zero among the patients with hormone receptor‐positive breast cancer are summarized in Table 1. There were significantly more invasive ductal carcinoma (97.3% vs. 95.0%) and less invasive lobular carcinoma (1.0% vs. 4.0%) in the HER2‐low group compared to the HER2‐zero group (*p* = 0.019). There was no significant difference (*p* > 0.05) between the HER2‐low group and HER2‐zero group in terms of age, histological grade, T stage, N stage, TNM stage, lymphovascular invasion, ki‐67, endocrine treatment, and radiotherapy. In general, the clinicopathological features of HER2‐low and HER2‐zero were balanced.

**TABLE 1:** Clinical and pathological characteristics of the HER2‐low group and the HER2‐zero group.

Characteristic	HER2‐zero (*n* = 200)	HER2‐low (*n* = 597)	*p*‐value
Median age (years)	47.3	48.8	0.092
Age (years)			0.766
≤40	45 (22.5%)	127 (21.3%)	
>40	155 (77.5%)	470 (78.7%)	
Histological type			0.019
Ductal	190 (95.0%)	581 (97.3%)	
Lobular	8 (4.0%)	6 (1.0%)	
Others	2 (1.0%)	10 (1.7%)	
Histological grade			0.937
1	9 (4.5%)	29 (4.9%)	
2	136 (68.0%)	408 (68.3%)	
3	35 (17.5%)	115 (19.3%)	
NA	20 (10.0%)	45 (7.5%)	
T stage			0.146
T1	91 (45.5%)	323 (54.1%)	
T2	99 (49.5%)	247 (41.4%)	
T3	9 (4.5%)	21 (3.5%)	
T4	1 (0.5%)	6 (1.0%)	
N stage			0.734
N1	105 (52.5%)	327 (54.8%)	
N2	60 (30.0%)	162 (27.1%)	
N3	35 (17.5%)	108 (18.1%)	
TNM stage			0.683
2	103 (51.5%)	318 (53.3%)	
3	97 (48.5%)	278 (46.6%)	
NA	0 (0%)	1 (0.2%)	
Lymphovascular invasion			0.284
No	147 (73.5%)	413 (69.2%)	
Yes	53 (26.5%)	184 (30.8%)	
Ki‐67			0.480
<20%	75 (37.5%)	219 (36.7%)	
≥20%	100 (50.0%)	335 (56.1%)	
NA	25 (12.5%)	43 (7.2%)	
Endocrine therapy			0.300
SERM	62 (31.0%)	159 (26.6%)	
NSAI	43 (21.5%)	145 (24.3%)	
SAI	16 (8.0%)	63 (10.6%)	
NA	79 (39.5%)	230 (38.5%)	
Radiotherapy			0.169
No	32 (16.0%)	128 (21.4%)	
Yes	129 (64.5%)	374 (62.6%)	
NA	39 (19.5)	95 (15.9%)	

*Note*: NA's are not included into difference analysis.

Abbreviations: NSAI, nonsteroidal aromatase inhibitor; SAI, steroid aromatase inhibitors; SERM, selective estrogen receptor modulator.

### Survival analysis

**FIGURE 1:**
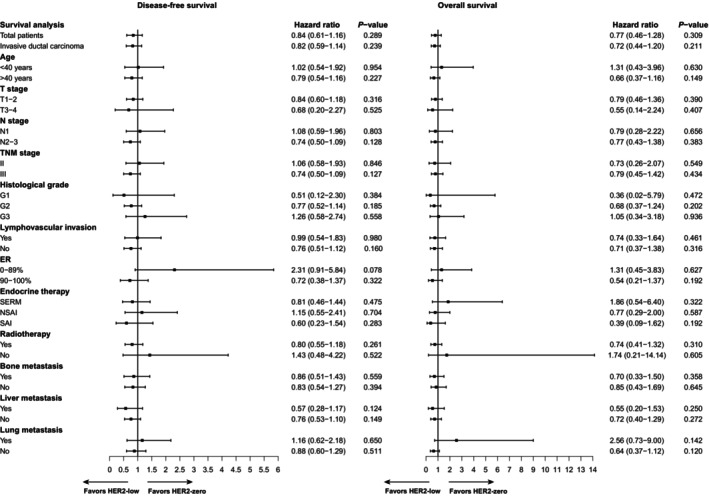
Disease‐free survival and overall survival of the HER2‐low group and the HER2‐zero group by Cox regression analysis. The left part is the comparison of disease‐free survival and the right part is the comparison of overall survival. ER, estrogen receptor; SERM, selective estrogen receptor modulator; NSAI, nonsteroidal aromatase inhibitor; SAI, steroid aromatase inhibitors.

**FIGURE 2:**
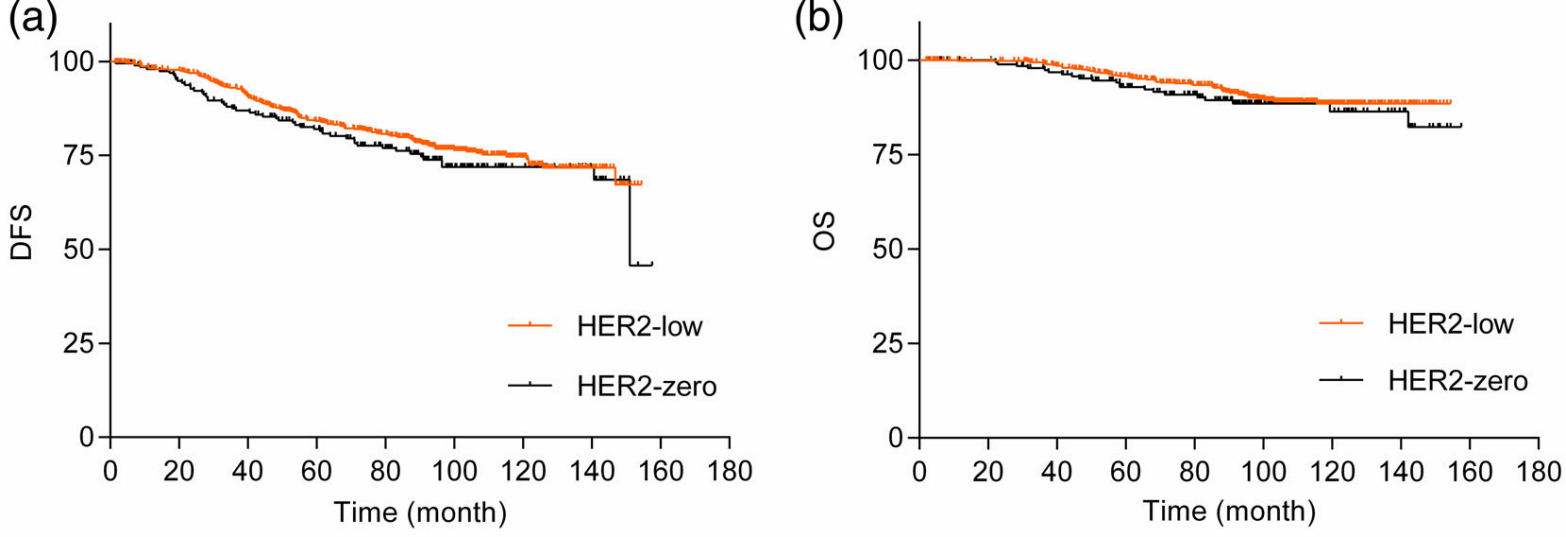
Disease‐free survival and overall survival of the HER2‐low group and the HER2‐zero group by Kaplan–Meier curves: (a) disease‐free survival, DFS (*p* = 0.288); (b) overall survival, OS (*p* = 0.307). DFA, disease‐free survival; OS, overall survival.

Among the hormone receptor‐positive group, the estimated rate of 10‐year disease free survival (DFS) were 74.70% in the HER2‐low group and 71.90% in the HER2‐zero group. The estimated rate of 10‐year overall survival (OS) was also 88.50% in the HER2‐low group and 86.50% in the HER2‐zero group. Through the univariate Cox regression analysis, similar DFS were observed between the HER2‐low group and HER2‐zero group (HR 0.84, 95% CI: 0.61–1.16, *p* = 0.290). In addition, there was no significant difference in OS between the HER2‐low group and HER2‐zero group (HR 0.77, 95% CI: 0.46–1.28, *p* = 0.310) through the univariate Cox regression analysis (Figure 1). The survival estimated by the Kaplan–Meier curves also showed no significant difference in DFS (*p* = 0.288, Figure 2a) and OS (*p* = 0.307, Figure 2b) between the HER2‐low group and the HER2‐zero group.

According to the subgroup of invasive ductal carcinoma, whether the age is over 40 years old or not, T stage, N stage, TNM stage, histological grade, lymphovascular invasion, estrogen receptor (ER), endocrine therapy, radiotherapy, bone metastasis, liver metastasis, and lung metastasis, there was no significant difference between the HER2‐low group and HER2‐zero group in DFS (*p* > 0.05) and OS (*p* > 0.05) (Figure 1).

**FIGURE 3:**
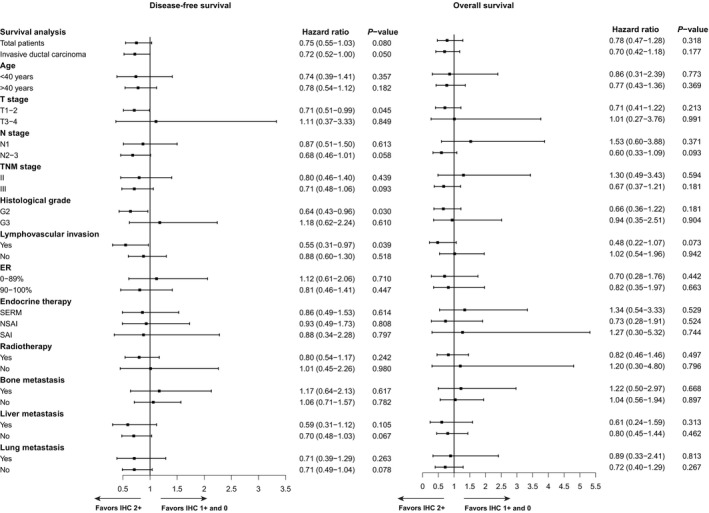
Disease‐free survival and overall survival of the IHC 2+ group and the IHC 1+ and 0 group by Cox regression analysis. IHC 1+ and IHC 0 were regarded as a group to be compared with IHC 2+. The left part is the comparison of disease‐free survival and the right part is the comparison of overall survival. ER, estrogen receptor; SERM, selective estrogen receptor modulator; NSAI, nonsteroidal aromatase inhibitor; SAI, steroid aromatase inhibitors.

It is worth noting that previous studies have shown that HER2 IHC 1+ and IHC 0 are often difficult to distinguish in immunohistochemistry. The consistency rate of judgments of HER2 IHC 1+ and IHC 0 by different pathologists is low.^11^ Therefore, in this study, considering that HER2 IHC 1+ and IHC 0 may inevitably have misjudgments in clinical practice, we creatively tried to take IHC 1+ and IHC 0 as a group to eliminate the impact of potential pathological misjudgments. No significant differences were observed in the total patients in DFS or OS between the IHC 2+ group and the IHC 1+ and IHC 0 group (Figure 3). Subgroup analyses were performed according to histological type, whether the age is over 40 years old or not, T stage, N stage, TNM stage, histological grade, lymphovascular invasion, ER, endocrine therapy, radiotherapy, bone metastasis, liver metastasis, and lung metastasis. We found the DFS of the HER2 IHC 2+ group was significantly better than that of the HER2 IHC 1+ and IHC 0 group in the invasive ductal carcinoma subgroup (HR 0.72, 95% CI: 0.52–1.00, *p* = 0.050), the T1–T2 subgroup (HR 0.71, 95% CI: 0.51–0.99, *p* = 0.045), the grade 2 subgroup (HR 0.64, 95% CI: 0.43–0.96, *p* = 0.030), and the lymphovascular invasion subgroup (HR 0.55, 95% CI: 0.31–0.97, *p* = 0.039) (Figure 3).

### Metastatic sites

**FIGURE 4:**
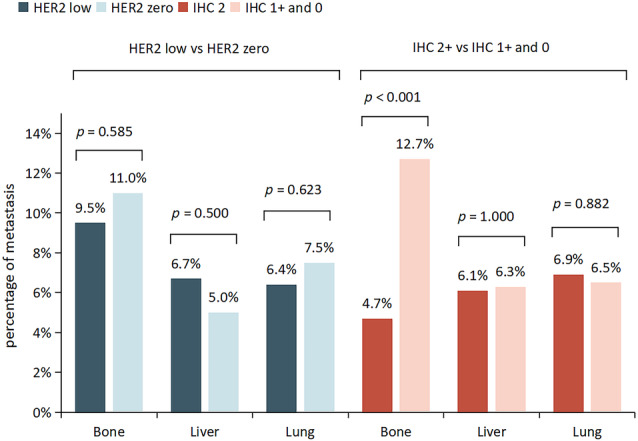
Metastatic sites of the HER2‐low group and the HER2‐zero group. Percentage of metastasis is the proportion of patients with metastasis to the total number of patients in each HER2 subgroup. If a patient has two or more metastatic sites, such as both bone metastasis and liver metastasis, then the incidence of both bone metastasis and liver metastasis will be calculated for the patient. The abscissa is the metastatic sites.

For the HER2‐low group of 597 patients, the most frequent metastatic sites were bone, liver, and lung in which there were 57 (9.5%) patients with bone metastasis, 40 (6.7%) patients with liver metastasis, and 38 (6.4%) patients with lung metastasis. For the HER2‐zero group of 200 patients, the most frequent metastatic sites were bone, lung, and liver, in which there were 22 (11.0%) patients with bone metastasis, 15 (7.5%) patients with lung metastasis, and 10 (5.0%) patients with liver metastasis. There were no significant differences in the incidence of bone metastasis, liver metastasis and lung metastasis between the HER2‐low group and the HER2‐zero group (*p* > 0.05) (Figure 4).

Similarly, we took HER2 IHC 1+ and IHC 0 as a group to be compared with HER2 IHC 2+ group. We also found that the most frequent metastatic sites were lung (6.9%), liver (6.1%), and bone (4.7%) in the HER2 IHC 2+ group, while in the HER2 IHC 1+ and IHC 0 group, the most frequent metastatic sites were bone (12.7%), lung (6.5%), and liver (6.3%), respectively. For bone metastasis, only 4.7% of the patients in the HER2 IHC 2+ group developed bone metastasis, while it occurred in 12.7% of the patients in the HER2 IHC 1+ and IHC 0 group. The incidence of bone metastasis in the HER2 IHC 2+ group was significantly lower than that in the HER2 IHC 1+ and IHC 0 group. In addition, there was no significant difference in the incidence of liver metastasis and lung metastasis between the HER2‐low group and the HER2‐zero group (*p* > 0.05) (Figure 4).

### Time curve: Recurrence rate and mortality

**FIGURE 5:**
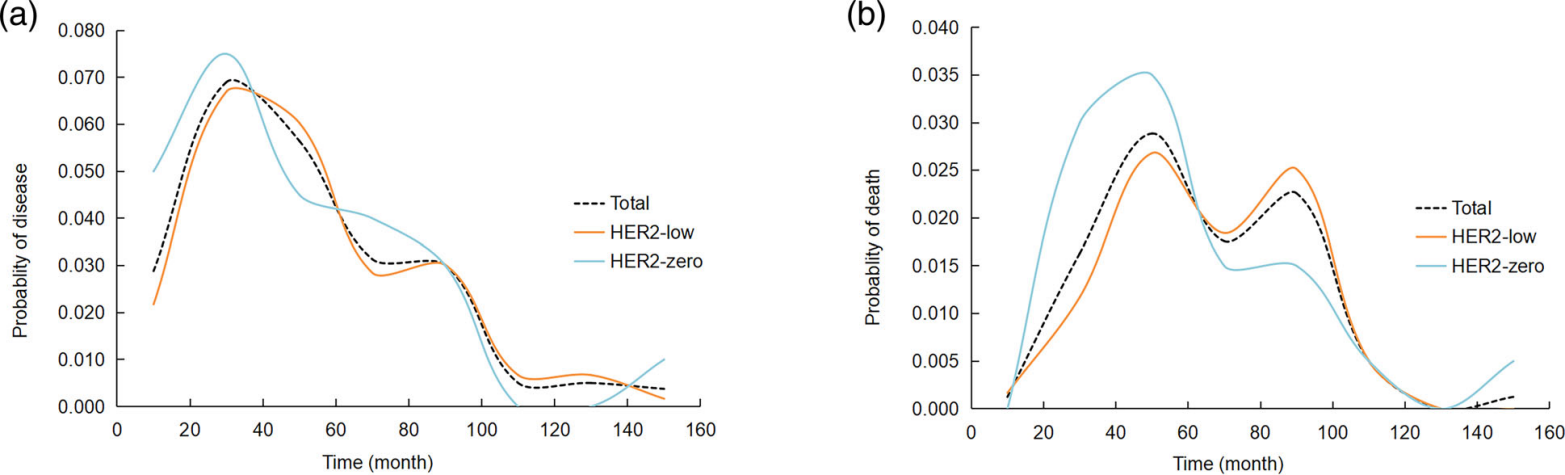
Time curve of the relapse rate and mortality in the total patients and the HER2‐low group and the HER2‐zero group: (a) probability of disease; (b) probability of death.

In both the HER2‐low group and the HER2‐zero group, recurrence rates continue to increase with time, reaching a peak relapse within the 20–40th month after surgery. In the 0–20th month, the recurrence rate of the HER2‐low group was lower than that of the HER2‐zero group, which was 2.2% and 5.0%, respectively. Within the 20–40th month, recurrence rates were similar in the HER2‐low group and the HER2‐zero group, 6.7% and 7.5%, respectively. The recurrence rate in the HER2‐zero group generally declined gradually from the 40–60th month after surgery, while the HER2‐low group still had a peak of recurrence between the 80–100th month. In the HER2‐low group, the recurrence rate was 2.8% in the 60–80th month and 3.0% in the 80–100th month (Figure 5a).

In both the HER2‐low group and the HER2‐zero group, the mortality continued to increase with time after surgery, reaching a peak within the 40–60th month after surgery, which is compatible with the peak in recurrence within the 20–40th month. In the 0–60th month, the HER2‐low group had a lower mortality rate than the HER2‐zero group. The HER2‐low group had a lower mortality rate than the HER2‐zero group in the 40–60th month following the peak of deaths, 2.7% versus 3.5%, respectively. After the 60th month, the trend began to reverse. After the 60th month, the HER2‐low group had an overall higher mortality rate than the HER2‐zero group. Notably, mortality in the HER2‐zero group declined gradually after the 60th month, while the HER2‐low group still had a small peak in deaths at the 80–100th month, which is also compatible with the peak in recurrence in the HER2‐low group at the 80–100th month. In the 80–100th month, the HER2‐low group had a higher mortality rate than the HER2‐zero group, which was 2.5% and 1.5%, respectively (Figure 5b).

## DISCUSSION

In our study, there was no significant difference in OS and DFS between the HER2‐low group and the HER2‐zero group. This may be related to the fact that all of the breast cancers in our study were hormone receptor‐positive. Recently, a large‐scale multiomics study^12^ has shown that the difference between HER2 IHC 1+ and IHC 2+ breast cancer in the hormone receptor‐negative subgroup was more significant than in the hormone receptor‐positive subgroup at the levels of the transcriptome, proteome, and metabolome. In addition, compared with the hormone receptor‐negative subgroup, the molecular characteristics of breast cancer with HER2‐low and HER2‐zero were more similar in the hormone receptor‐positive subgroup.^12^ This may explain why there was no significant difference in prognosis between the HER2‐low and HER2‐zero groups in our study.

In addition, just like other existing studies studying the difference in prognosis between the HER2‐low group and the HER2‐zero group, it is inevitable that there may be a misdiagnosis between HER2 IHC 1+ and IHC 0. In current clinical practice, HER2 IHC 2+ can represent HER2‐low more accurately, and it is more appropriate to combine HER2 IHC 1+ and IHC 0 into one group due to the difficulty in distinguishing them in clinical practice. Therefore, we propose to take HER2 IHC 1+ and IHC 0 as a group, and we found that the DFS of the HER2 IHC 2+ group was significantly better than that of HER2 IHC 1 and IHC 0 group in some subgroups such as the invasive ductal carcinoma subgroup, the T1–T2 subgroup, grade 2 subgroup, and lymphovascular invasion subgroup.

Since the purpose of HER2 IHC identification in the past was to indicate subsequent HER2 targeted therapy, the main focus was on distinguishing positive from negative, rather than HER2 IHC 0 and HER2 IHC 1+. As mentioned earlier, it is reported that for HER2 IHC in the low range (i.e., IHC 0 and IHC 1+), the agreement between different pathologists was only 26%.^11^ In the phase 1b study of T‐DXd, the agreement rate of HER2 IHC 2+ was 40%, and the agreement rate of HER2 IHC 1+ was 70% between the local and central pathologists.^13^ The pathological diagnosis of HER2 has entered the era of three classifications nowadays, but the consistency of HER2‐low interpretation among different pathologists is low in practice. In recent years, artificial intelligence (AI) had great progress in identifying tumor cells through machine learning immunohistochemical pictures. With the continuous improvement and maturity of technology, AI may give us important help in the diagnosis of HER2‐low.

To the best of our knowledge, this is the first study to compare the metastatic sites of HER2‐low and HER2‐zero in hormone receptor‐positive breast cancer. According to our study, the most frequent metastatic sites in the HER2‐low group were bone (9.5%), liver (6.7%), and lung (6.4%). In the HER2‐zero group, the most frequent metastatic sites were bone (11.0%), lung (7.5%), and liver (5.0%). Bone (especially axial bone) is the most common site for breast cancer metastasis.^14^ About 50% of advanced breast cancer patients have bone metastasis.^15^ The incidence of bone metastasis in total patients of the HER2 IHC 1+ and IHC 0 group (12.7%) was significantly higher than that in the HER2 IHC 2+ group (4.7%) (*p* < 0.001), which suggested that for patients in the HER2 IHC 1+ and IHC 0 group, early screening of bone metastasis in breast cancer patients is important for early detection and early treatment of bone metastasis. According to the previous study, if the bone‐modifying agents (BMAs) are not used in time after bone metastasis in breast cancer patients, about 30% of patients will have skeletal‐related events (SREs) within 3 months.^16^ Meanwhile, SREs caused by bone metastasis, such as pathological osteolysis, spinal compression, pathological fracture, and hypercalcemia, seriously affect patients' quality of life.^17^ Not only is the quality of life decreased after bone metastasis, but survival is also significantly shortened.^18^ According to the statistics of the American Cancer Society (ACS), the 5‐year survival rate of breast cancer patients is 91%, and the 10‐year survival rate is 84%.^19^ However, the survival time of breast cancer patients with bone metastasis is significantly shortened, and the 3‐year survival rate of breast cancer patients with bone metastasis is only 50.5%, and the median survival time is 36 months.^20^ Hence, for patients with HER2 IHC 1+ and IHC 0 in hormone receptor‐positive breast cancer, early screening of bone metastasis and early treatment are helpful to improve the quality of life and prognosis of patients. Tumor cells invade locally to escape the surrounding tissues of the primary tumor, then invade the blood or lymphatic vessels as circulating tumor cells to survive in the circulatory system, then escape from the circulatory system, and adapt to the microenvironment as disseminated tumor cells (DTCs) to become metastasis. We hypothesized that different types of breast cancer cells have different ability to colonize in the bone microenvironment, so the incidence of bone metastasis is different.

In this study, we analyzed the time curve of recurrence and mortality. The HER2‐low group had a lower mortality rate than the HER2‐zero group before postoperative 40–60th month. After postoperative 60th month, the trend began to reverse. After the 60th month, the HER2‐low group had an overall higher mortality rate than the HER2‐zero group. Compared with the HER2‐zero group, we found that the HER2‐low group had a more obvious peak in death at the time of postoperative 80–100th month. This suggested that patients with HER2‐low should pay more attention to the long‐term recurrence risk, and regular review and monitoring are needed.

Some studies have come to the conclusion that among hormone receptor‐positive breast cancer, HER2‐low had significantly better survival than HER2‐zero.^8^, ^21^, ^22^, ^23^ It has been suggested^24^ that the better prognosis of the HER2‐low breast cancer may be related to the higher expression of Luminal related genes in the HER2‐low breast cancer, as this improves the response of the HER2‐low breast cancer to endocrine therapy. The difference between the HER2‐low and HER2‐zero breast cancer may be related to different hormone receptor expression levels. It has been previously reported that after adjusting for hormone receptor expression, there was no significant molecular difference between HER2‐low and HER2‐zero breast cancer.^6^ Therefore, it has been suggested^25^ that HER2‐low breast cancer should not be considered as a distinct molecular subtype.

However, in another study, no significant differences in luminal‐associated gene expression or higher endocrine sensitivity scores were observed in hormone receptor‐positive HER2‐low breast cancer.^12^ Lower levels of 17q21.31 and 17q11.2 loss/deletion were also observed in HER2‐low breast cancer than in HER2‐zero breast cancer, which was correlated with a better prognosis in hormone receptor‐positive patients.^12^ Different studies have reached different conclusions as to whether there is a prognostic difference between the HER2‐low group and the HER2‐zero group in breast cancer. A previous meta‐analysis of 14 studies found that among patients with early‐stage hormone receptor‐positive BC, compared to the HER2‐zero BC, the HER2‐low BC had significantly favorable OS, DFS, and relapse‐free survival (RFS).^26^

Whether HER2‐low is an independent subtype is still controversial. Some studies have shown that the TP53 mutation rate is lower in the HER2‐low group than the HER2‐zero group.^6^ Genomics studies have also found that the biological behavior of the HER2‐low group is actually driven by hormone receptor status.^24^ By comparing the gene mutation profiles of breast cancer, it was found that the mutations of CBFB, PIK3CA, MAP3K1, and ARID1A in the HER2‐low subgroup were more common, while the mutations in TP53, TERT, GALNT12, CARD11, TRRAP in the HER2‐zero subgroup were more common.^27^ Breast cancer is a highly heterogeneous malignant tumor.^3^ Also, there is heterogeneity in the HER2‐low breast cancer. A cohort^12^ of 434 Chinese patients with HER2‐low breast cancer with multiomics data has shown that HER2‐low is more distinct from HER2‐zero in triple negative breast cancer (TNBC) compared with hormone receptor‐positive breast cancer. Basal‐like breast cancer is like HER2‐zero breast cancer, and non‐basal‐like HER2‐low breast cancer is like HER2‐positive breast cancer in molecular characteristics. It is important to realize the heterogeneity of HER2‐low breast cancer and precise stratification regarding hormone receptor status and molecular subtype is needed. Nowadays, endocrine therapy is important in hormone receptor‐positive/HER2‐negative advanced breast cancer.^28^ Recently, with the study of ADC in breast cancer with HER2‐low, novel ADC drugs have become an important choice for the treatment of advanced breast cancer with HER2‐low.^29^ However, the use of ADCs in advanced HER2‐low breast cancer, such as the order of use of HER2‐targeted ADCs and TROP2‐targeted ADCs, is still controversial and needs to be verified by more clinical trials.^30^

In conclusion, through an analysis of 797 patients with hormone receptor‐positive breast cancer, this study compared recurrence patterns in the HER2‐low group and the HER2‐zero group. Compared with the HER2‐zero group, we found that the HER2‐low group had a more obvious peak in mortality at the time of postoperative 80–100th month, which suggested the importance of long‐term monitor for patients with HER2‐low in hormone receptor‐positive breast cancer. For patients with HER2 IHC 1+ and 0, the risk of bone metastasis is significantly higher than that of patients with HER2 IHC 2+, which suggested that patients with HER2 IHC 1+ and 0 should pay more attention to the review and monitoring of bone metastasis. In addition, there was no significant difference in DFS and OS between the HER2‐low group and the HER2‐zero group in this study. When IHC 1+ and 0 are taken as a group, the IHC 2+ group has significantly better DFS than the IHC 1+ and 0 group in some subgroups.

## AUTHOR CONTRIBUTIONS

Concept and design, drafting and critical revison of the manuscript: Peng Yuan and Tong Wei. Acquisition, analysis, or interpretation of data: Tong Wei, Yikun Kang, Xue Wang, Jian Yue, Binghe Xu and Peng Yuan.

## FUNDING INFORMATION

Beijing Medical Award Foundation (YXJL‐2020‐0941‐0763), Chinese Academy of Medical Sciences Clinical Translational and Medical Research Fund (2022‐I2M‐C&T‐A‐014).

## CONFLICT OF INTEREST STATEMENT

The authors declare no conflicts of interest.
